# Eosinophils Control Liver Damage by Modulating Immune Responses Against *Fasciola hepatica*

**DOI:** 10.3389/fimmu.2020.579801

**Published:** 2020-09-18

**Authors:** Sofía Frigerio, Valeria da Costa, Monique Costa, María Florencia Festari, Mercedes Landeira, Santiago A. Rodríguez-Zraquia, Steffen Härtel, Jorge Toledo, Teresa Freire

**Affiliations:** ^1^Laboratorio de Inmunomodulación y Desarrollo de Vacunas, Departamento de Inmunobiología, Facultad de Medicina, Universidad de La República, Montevideo, Uruguay; ^2^Laboratorio de Análisis Imágenes Científicas, SCIAN-lab, Instituto de Neurociencias Biomédicas (BNI), Facultad de Medicina Universidad de Chile, Santiago, Chile

**Keywords:** *Fasciola hepatica*, eosinophils, immunomodulation, antibodies, degranulation, antibody-dependent cell cytotoxicity

## Abstract

Eosinophils are granulocytes that participate in the defense against helminth parasites and in hypersensitivity reactions. More recently, eosinophils were shown to have other immunomodulatory functions, such as tissue reparation, metabolism regulation, and suppression of Th1 and Th17 immune responses. In the context of parasitic helminth infections, eosinophils have a controversial role, as they can be beneficial or detrimental for the host. In this work, we investigate the role of eosinophils in an experimental infection in mice with the trematode parasite *Fasciola hepatica*, which causes substantial economical losses around the world due to the infection of livestock. We demonstrate that eosinophils are recruited to the peritoneal cavity and liver from *F. hepatica*-infected mice and this recruitment is associated with increased levels of CCL11, TSLP, and IL-5. Moreover, the characterization of peritoneal and hepatic eosinophils from *F. hepatica*-infected mice showed that they express distinctive molecules of activation and cell migration. Depletion of eosinophils with an anti-Siglec-F antibody provoked more severe clinical signs and increased liver damage than control animals which were accompanied by an increase in the production of IL-10 by hepatic and splenic CD4^+^ T cells. In addition, we also report that eosinophils participate in the modulation of humoral immune responses during *F. hepatica* infection, contributing to their degranulation. In conclusion, we demonstrate that eosinophils are beneficial for the host during *F. hepatica* infection, by limiting the production of IL-10 by specific CD4^+^ T cells and favoring eosinophil degranulation induced by specific antibodies. This work contributes to a better understanding of the role of eosinophils in parasitic helminth infections.

## Introduction

*Fasciola hepatica*, a worldwide-distributed liver fluke, is one of the causative agents of fasciolosis, a zoonotic disease that affects livestock and humans (1). Indeed, the World Health Organization estimates that around 17 million people are infected and 180 million are at risk of infection, predominantly in South America and Africa (2). In addition, fasciolosis causes huge economical losses due to livestock infection, of approximately 3 billion dollars per year (1). The life cycle of *F. hepatica* is complex as the parasite goes through multiple stages before reaching its adult form and includes an intermediate host, a water snail of the *Lymnaea* genus, and a definitive host, usually livestock or humans (3). After ingestion of metacercariae by the mammalian host, juvenile flukes penetrate the host’s intestine wall and reach the liver between 4 and 6 days. Eventually, the flukes reach the bile ducts, where they become sexually mature (3). *F. hepatica* causes chronic infections due to sophisticated immune modulation strategies that make possible its long-period survival in the host. Indeed, several studies have shown that the parasite induces regulatory dendritic cells (4, 5), alternative activated macrophages (6), and a type 2 modified immune response characterized by an important regulatory T cell (Treg) component (4, 7).

Eosinophils, granulocytes belonging to the innate immune system, participate mainly in the defense against multicellular parasites and in several Th2-driven immune disorders, such as asthma, atopic dermatitis, and eosinophilic esophagitis (8). The classical functions of eosinophils include mainly degranulation triggered by antibodies in a mechanism known as antibody-dependent cell cytotoxicity (ADCC) (8, 9). However, in the last years, novel immunomodulatory functions have been reported such as the regulation of glucose metabolism in the adipose tissue (10), enhancement of plasma cells survival (11), and suppression of Th1 immune responses (12, 13).

Eosinophils play a pivotal role in fighting against some helminth infections (9), reflected by their dramatic increase in response to IL-5 produced by CD4^+^ type 2 helper T cells (Th2) (14). Although their capacity for killing helminths through ADCC *in vitro* has been well demonstrated (15, 16), their role *in vivo* remains controversial. While in some cases eosinophils appear to be beneficial for the host (17) in others their presence is redundant (18) or even of apparent benefit for the parasite (19). In *F. hepatica* infection, however, the role of eosinophils remains unknown.

In this study we characterized eosinophils during experimental infection with *F. hepatica* in mice and demonstrate that they contribute to limit liver damage induced by the parasite by reducing the production of IL-10 by CD4^+^ T cells. In addition, our results indicate that eosinophils play a role in specific humoral immunity by inducing the production of more effective antibodies in triggering eosinophil degranulation. Thus, the present study contributes to the elucidation of immunomodulatory mechanisms mediated by eosinophils during *F. hepatica* infection and collaborates with the understanding of their role in helminth infections.

## Materials and Methods

### Ethics Statement

Mouse experiments were carried out in accordance with strict guidelines from the National Committee on Animal Research (Comisión Nacional de Experimentación Animal, CNEA, http://www.cnea.gub.uy/, National Law 18.611, Uruguay) according to the international statements on animal use in biomedical research from the Pan American Health Organization (PAHO) and World Health Organization (WHO). The protocol was approved by the Uruguayan Committee on Animal Research. Cattle’s livers were collected during the routine work of a local abattoir (Frigorífico Carrasco) in Montevideo (Uruguay).

### Mice

Six- to eight-week-old female BALB/c mice were purchased from DILAVE Laboratories (Uruguay). Animals were kept in the animal house (URBE, Facultad de Medicina, UdelaR, Uruguay) with water and food supplied *ad libitum*. Mouse handling and experiments were carried out in accordance with strict guidelines from the National Committee of Animal Research (CNEA, Uruguay). All procedures involving animals were approved by UdelaR’s Committee on Animal Research (CHEA, Protocol Number: 070153-000820-17).

### Preparation of Protein Lysates From *F. hepatica*

Live adult worms of *F*. *hepatica* were obtained from the bile ducts of bovine livers, washed in phosphate-buffered saline (PBS) pH 7.4, then mechanically disrupted and sonicated. After centrifugation at 40,000 × *g* for 60 min, supernatants were collected and dialyzed against PBS. The obtained lysate (FhTE) was quantified and stored at -80°C. The endotoxin levels were determined by using the Limulus Amebocyte Lysate kit Pyrochrome (Associates of Cape Cod). Adult worms were collected during the routine work of a local abattoir (Frigorífico Carrasco and Sarubbi) in Montevideo (Uruguay). Protocols were approved by the Uruguayan Committee on Animal Research (CHEA, Protocol Number: 070153-000820-17).

### *F. hepatica* Infections

BALB/c mice were orally infected with 10 *F. hepatica* metacercariae (Montevideo, Uruguay) per animal. After 8, 15, or 21 days post-infection (d.p.i.) mice were bled and peritoneal exudate cells (PECs), spleens, and livers were removed. Non-infected animals were used as controls. At least 4 animals were used per group. Peritoneal exudate cells were harvested by washing the peritoneal cavity with 10 ml of cold PBS. For the isolation of hepatic leukocytes, a previously described protocol was followed (18). Briefly, livers were mechanically dissociated and the cell suspension was left on ice for 15-20 min. The supernatant was then recovered in a new tube and centrifuged at 120 × *g* for 7 min at 4°C. The pellet was resuspended in 40% Percoll gradient and centrifuged at 600 × *g* for 20 min at 20°C, after which the hepatic leukocytes were obtained. Finally, red cells were lysate and hepatic leukocytes were washed with PBS.

In order to deplete eosinophils, mice (*n* = 7–8/group) received an intraperitoneal injection of 15 μg of monoclonal rat IgG_2*a*_ anti-Siglec-F antibody (clone E50-2440 from BD Biosciences, United States), the day before and after infection with *F. hepatica* and every 3 days until sacrifice. The control group consisted of mice injected intraperitoneally with isotype-matched control antibody (BD Biosciences, United States). Mice were sacrificed at day 20 post-infection, bled, and PECs, spleens and livers were removed and used for the following experiments. The severity of the infection was assessed by determining the general state of the animal by a defined clinical score according to the following parameters: peritoneal hemorrhage, macroscopic liver damage, splenomegaly, and cell content in the peritoneal cavity (20), where the minimum score was 0 whereas maximal score was 10. Liver damage was quantified by testing alanine aminotransferase (ALT) activity in sera, determined by using a commercial kit (Spinreact, Spain) according to the manufacturers’ instructions.

### Flow Cytometry

Obtained cell suspensions from PECs, livers and spleens were washed twice with PBS containing 2% FBS and 0.1% sodium azide (FACS buffer) and stained with specific antibodies for 30 min at 4°C. The following antibodies were used in these experiments: anti-Siglec-F ((E50-2440 and S17007L), -F4/80 (BM8), -CD11b (M1/70), -Sirpα (P-84), -CCR3 (J073E5), -Ly6G (RB6-8C5), -Ly6C (HK1.4), -CD44 (IM7), -CD162 (2PH1), -CD4 (GK.1), -CD8 (53-6.7), and -I-A/I-E (M5/114.15.2). Cells were then washed twice with FACS buffer and fixed with 0.1% formaldehyde in PBS for 15 min. Expression of FoxP3, IL-4, IFN-γ, and IL-10 was analyzed by intracellular staining by incubating cells in Brefeldin-A for 6 h at 37°C in the presence of phorbolmyristate acetate (PMA, 200 nM) (Sigma Aldrich, United States). They were then washed twice with wash buffer and incubated with the following antibodies: anti-CD3 (17A2), -CD4 (RM4-5), -CD8 (53-6.7). After permeabilization with Cytofix and Perm wash buffers (Biolegend, United States), cells were incubated with FoxP3 (FJK-16s), IL-4 (11B11), IFN-γ (XMG1.2), and IL-10 (JES5-1E3). Cell populations were analyzed using a BD Accuri C6 Plus (BD-Biosciences) or Cyan (Beckman Coulter). Antibodies were obtained from BD-Biosciences (CA, United States) or from Biolegend (CA, United States). Analyses were performed with Flowjo V10 software.

### Sorting of Eosinophils

Peritoneal exudate cells from infected mice after 18–20 d.p.i. were obtained and stained with antibodies against Siglec-F and F4/80 for 30 min at 4°C. Eosinophils (Siglec-F^+^ F4/80^*int*^) were sorted at 4°C in a BD FACSAria Fusion Cell Sorter. The purity and viability of the sorted eosinophils was >90%.

### Histology

Livers from infected mice after 21 d.p.i. or non-infected mice (control) were harvested, embedded in Polyfreeze (Sigma-Aldrich, United States), and snap-frozen in nitrogen. Sections were cut at a thickness of 10 μm, stained with hematoxylin and eosin and analyzed under light microscope Zeiss Axio LabA1.

### Immunofluorescence

Peritoneal exudate cells and sorted eosinophils from infected mice were dispersed in silanized slides. Cells were permeabilized with 0.1% Triton X-100 in PBS for 15 min and blocked with 5% BSA for 1 h at room temperature. Cells were then overnight incubated at 4°C with anti-Siglec-F (E50-2440), -F4/80 (BM8), -CD11b (M1/70), -CCR3 (J073E5), -Ly6G (HK1.4), -CD11c (N418), -CD44 (IM7), -I-A/I-E (M5/114.15.2), -CCR2 (SA203G11), and -CD162 (2PH1), stained with 4’,6-diamidino-2-phenylindole and visualized in a confocal microscope Leica TCS-SP5-II, Olympus XI 81 Spinning Disk and Leica TCS LSI. For May-Grünwald Giemsa staining, cells were stained with May-Grunwald staining for 1 min, washed with distilled water, followed by Giemsa staining. Finally, cells were washed with distilled water and analyzed in a microscopere Nikon Eclipse E400 and Zeiss Axio Lab.A1.

### Proliferation Assay

Splenocytes (0.5 × 10^6^/well) from eosinophil depleted- and control mice were obtained and cultivated in RPMI-1640 with glutamine (Capricorn, Gibco, Germany) complete medium containing 10% heat-inactivated fetal bovine serum (FBS, Capricorn Scientific, Germany), 50 μM 2-mercaptoethanol, 100 U/mL penicillin, 0.1 mg/mL streptomycin (Sigma-Aldrich, United States) in presence or absence of FhTE (75 μg/mL) for 5 days at 37°C and 5% CO_2_. IFNγ, IL-4, IL-5, and IL-10 levels were quantified on culture supernatants. Uninfected naive animals were used as a control group.

### Determination of Cytokines and Chemokines

IFN-γ, IL-4, IL-5, IL-10, thymic stromal lymphopoietin (TSLP), IL-5, CCL11 levels in culture supernatants or ascites of infected and non-infected mice were quantified by interleukin-specific sandwich ELISA assays using the corresponding antibodies from BD Bioscience or Biolegend. TGFβ and FIZZ-1 were detected by real-time qRT-PCR using an Eco real-time PCR System (Illumina, United States) using Fast SYBR^®^ Green Master Mix (Applied Biosystems, United States). Total RNA was isolated from BALB/c mice treated with α-Siglec-F antibody or with isotype control, and from non-infected mice by using RNeasy Mini Kit (Qiagen, United States). Standard amplification conditions were 10 min at 95°C for initial activation, followed by 40 thermal cycles of 15 s at 95°C, 30 s at 60°C and 30 s at 72°C with a final extension of 10 min at 72°C. The following primers were used: TGFβ-F: 5′-AACAATTCCTGGCGTTACCTT-3′; TGFβ-R: 5′-CTGCCGTACAACTCCAGTGA-3′; GAPDH-F: 5′-CTGAGAACGGGAAGCTTG-3′; GAPDH-R: 5′-CCTGCT TCACCACCTTCTTG-3′; FIZZ1-F: 5′-CACCTCTTCACTCGA GGGACAGTTG-3′; FIZZ1-R: 5′-GGTCCCAGTGCATATGG ATGAGAC-3′. Results were expressed as the ratio between each evaluated cytokine and GAPDH expression. Expression was calculated using the 2-ΔΔCT method and normalized to GAPDH. All reactions were performed with at least five biological replicates.

### Determination of Specific *F. hepatica* Antibodies

To determine parasite-specific antibody titers in sera an indirect ELISA assay was performed. Briefly, 96-well microtiter plates (Nunc, Denmark) were coated with FhTE (2 μg/well) in 50 mM carbonate buffer (pH 9.6). After blocking with 1% gelatin in PBS, three washes with PBS containing 0.1% Tween-20 were performed. Serially diluted sera in PBS containing 0.1% Tween-20 and 0.5% gelatin were added to the wells for 1 h at 37°C. After three washes, wells were treated 1 h at 37°C using goat anti-mouse IgM, IgA, IgG IgG_1_, IgG_2*a*_, IgG_2*b*_, or IgG_3_ peroxidase-conjugates (Sigma-Aldrich, United States) followed by o-phenylenediamine (OPD) and H_2_O_2_ as substrates. Plates were read photometrically at 492 nm in an ELISA auto-reader (Thermo Fisher Scientific). Antibody titers were calculated to be the log_10_ highest dilution, which gave twice the absorbance of control (mock) mouse sera with the minor dilution. Titers are shown as the arithmetic mean ± SEM.

### Differentiation of Bone Marrow-Derived Eosinophils

Eosinophils were generated from cultures of bone marrow cells from infected and non-infected mice based on a previously described protocol (20). Briefly, bone marrow from the tibia and femur of BALB/c infected mice was used at 18 d.p.i. Cells were cultured at 0.5 × 10^6^/mL in complete RPMI medium supplemented with 100 ng/mL SCF and 100 ng/mL FLT3-L (PeproTech). On day 4, the media containing SCF and FLT3-L was replaced with media containing 10 ng/mL IL-5 (PeproTech) alone and cultured for 10 days at 37°C and 5% CO_2_.

### Degranulation of Eosinophils

Eosinophil degranulation was determined through the measurement of the eosinophil peroxidase (EPO) enzyme liberation, as previously described (21). Briefly, bone marrow-differentiated eosinophils were collected by centrifugation and resuspended in complete RPMI medium in a ninety-six-well microtiter culture plate (1 × 10^6^/well) in the presence or absence of sera from infected and non-infected mice (dilute 1/50) and FhTE (100 μg/mL) for 3 h at 37°C. The assay was developed using 100 μL OPD reagent (800 μL 15 mM OPD in 4 mL 1 M Tris (pH 8.0), 9.2 mL H_2_O and 4 μL 30% H_2_O_2_). The reaction was terminated by the addition of 100 μL of 3 M HCl to each well and read at 492 nm. Control conditions corresponded to untreated cells.

### Statistics

Experiments were performed as indicated and expressed as mean ± SEM. Statistical analyses were performed using GraphPad Prism version 6.04 for Windows (GraphPad Software, United States). Results were analyzed using one-way ANOVA followed by Tukey’s test, or two-tails student’s *t*-test. Significant differences were considered as follows: ^∗^*p* < 0.05; ^∗∗^*p* < 0.01; ^∗∗∗^*p* < 0.001; ^****^p < 0.0001.

## Results

### Eosinophils Are Recruited to the Peritoneal Cavity and Liver During *F. hepatica* Infection

**FIGURE 1:**
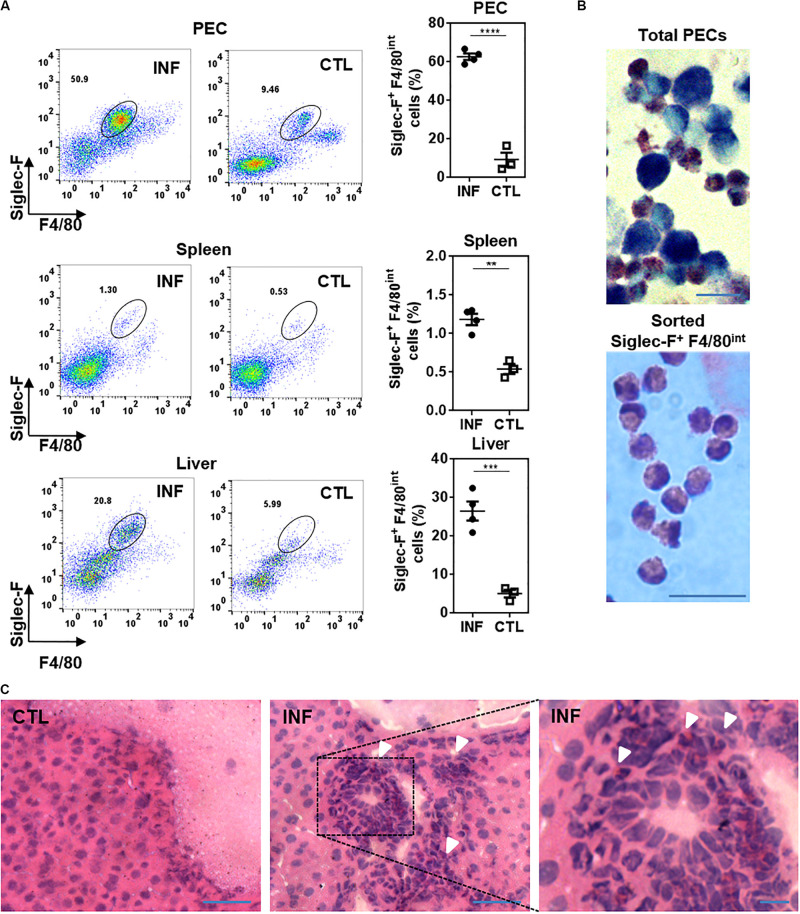
Eosinophils are recruited to the peritoneal cavity and liver from *F. hepatica*-infected animals. **(A)** PECs, spleens or livers were collected from 3-week infected mice and analyzed by flow cytometry after staining with anti-Siglec-F and -F4/80 specific antibodies. Asterisks correspond to significant differences as follows: *p < 0.05; **p < 0.01; ***p < 0.001; ****p < 0.0001, performed by student’s t-test. Each dot represents a single mouse. Infected and control mice are represented by circle and square symbols, respectively. **(B)** PECs from infected animals (upper image) or sorted peritoneal eosinophils (lower image) were stained with May-Grunwald Giemsa and analyzed by microscopy. The bars correspond to 10 μm. **(C)** Sections of livers stained with hematoxylin and eosin. CTL, control, INF, infected mice. The arrows show eosinophil-like cells. The bars correspond to 50 μm (left and center) or 10 μm (right).

We first evaluated whether Siglec-F^+^ cells were present in *F. hepatica*-infected animals. To this end, mice were infected with 10 metaceracariae and after 3 weeks post-infection (w.p.i.), livers, spleens and PECs were removed and eosinophils were analyzed by flow cytometry. These studies revealed that Siglec-F^+^ cells expressed intermediate levels of F4/80, thus they were identified as Siglec-F^+^ F4/80^*int*^ cells (Supplementary Figure 1A). These cells were detected in the peritoneal cavity, spleen and liver from infected animals. However, a recruitment of eosinophils both in PECs and liver was observed in *F. hepatica*-infected animals, while only a slight increase of these cells was found in the spleen (Figure 1A). Microscopy examination of May-Grunwald Giemsa stained PECs from infected animals showed a heterogenic group of cells comprising large cytoplasmic granules that stain purple and bi-lobulated nuclei typical of mouse eosinophils and cells with macrophage morphological features (Figure 1B, upper photo). Siglec-F^+^ F4/80^*int*^ cells were then sorted and microscopic examination confirmed that these cells were eosinophils (Figure 1B, lower photo). Last, granulocytic-like cells were also detected in livers from infected animals by their purple staining present in the leukocyte infiltrate of livers (Figure 1C).

**FIGURE 2:**
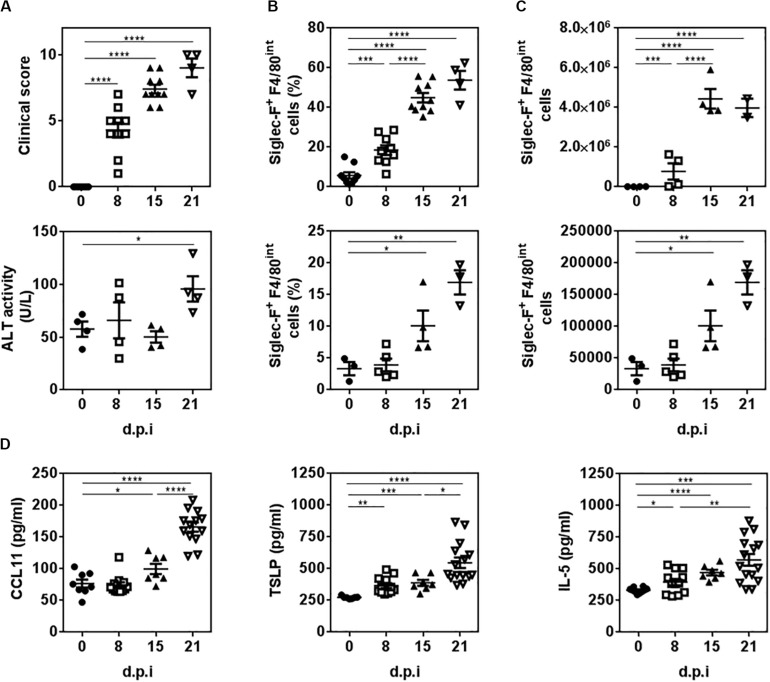
The recruitment of eosinophils to the peritoneal cavity and liver of infected animals is associated with increased levels of CCL11, TSLP and IL-5. Mice were infected with 10 metacercariae and sacrificed and 8, 15, and 21 days post-infection (d.p.i.). Non-infected mice were used as control (0 d.p.i.). **(A)** The severity of the disease was determined by the presence of clinical signs including hemorrhage, splenomegaly and macroscopic liver damage. ALT activity in sera was used to quantify liver damage. **(B)** Frequency and cell number of Siglec-F^+^ F4/80^*int*^ cells in the peritoneum of infected and animals by flow cytometry. **(C)** Frequency and cell number of Siglec-F^+^ F4/80^*int*^ cells in livers of infected and control mice by flow cytometry. **(D)** CCL11, TSLP and IL-5 levels in the peritoneal cavity of infected and control mice determined by specific sandwich ELISA. Asterisks correspond to significant differences as follows: *p < 0.05, **p < 0.01, ***p < 0.001, ****p < 0.0001, performed by one way ANOVA followed by Tukey’s test.

Then, we analyzed the presence of eosinophils during the experimental infection with *F. hepatica*. To this end, infected mice were sacrificed at 8, 15, and 21 d.p.i. to study the presence of eosinophils during the course of the infection. The analyses of infected mice revealed a progressive increase in the clinical signs (score) caused by the infection since day 8 post-infection, while liver damage was only detected at 21 d.p.i. evaluated by the ALT activity in serum (Figure 2A), a common marker to detect hepatic dysfunction (21). An increase of Siglec-F^+^ F4/80^*int*^ cells was detected in the peritoneal cavity of infected mice at 8 d.p.i. that was increased as infection progressed (Figure 2B and Supplementary Figure 2A). On the other hand, hepatic Siglec-F^+^ F4/80^*int*^ cells were detected only after 15 d.p.i. (Figure 2C and Supplementary Figure 2B) suggesting that the recruitment of eosinophils to the liver is a later event in the infection. Last, we analyzed the presence of important cytokines and chemokines for the recruitment or differentiation of eosinophils, such as CCL11, also known as eotaxin 1, TSLP and IL-5, both in the peritoneal cavity and serum. As shown in Figure 2D, TSLP, and IL-5 levels in the peritoneum increased at 8 d.p.i. while higher levels of CCL11 were found after 15 d.p.i. TSLP, CCL11, and IL-5 levels were highest at 21 d.p.i. On the other hand, non-detectable levels of these cytokines and chemokine were detected in sera from infected animals (Supplementary Figure 2C).

**FIGURE 3:**
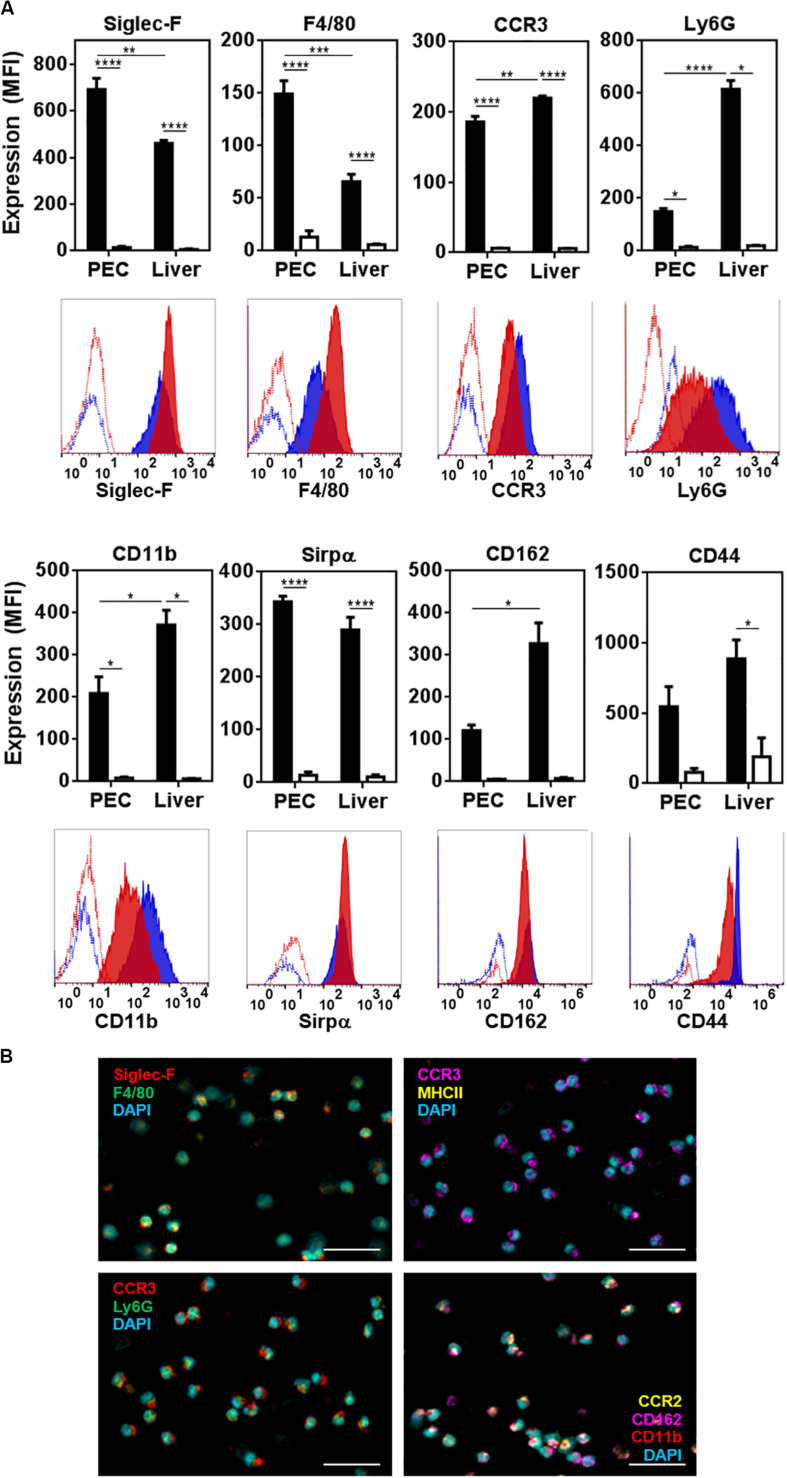
Characterization of peritoneal and hepatic eosinophils from *F. hepatica* infected mice. **(A)** The expression of Siglec-F, F4/80, CCR3, Ly6G, CD11b, Sirpα, CD162, and CD44 was determined on Siglec-F^+^ cells in the peritoneum (red histogram) or liver (blue histogram) from infected animals. Median fluorescence intensity is shown (MFI). Control corresponds to unstained cells (white bars). Asterisks correspond to significant differences as follows: *p < 0.05, **p < 0.01, ***p < 0.001, ****p < 0.0001, performed by one way ANOVA followed by Tukey’s test. **(B)** Expression of Siglec-F, F4/80, CCR3, Ly6G, CCR2, CD162, and CD11b in sorted peritoneal eosinophils (Siglec-F^+^ F4/80^*int*^ cells) by confocal microscopy. The bars correspond to 50 μm.

We then analyzed the molecules expressed by peritoneal and hepatic eosinophils. Flow cytometry and confocal microscopy analyses demonstrated that, apart from Siglec-F and F4/80, both types of eosinophils expressed CCR3, the receptor for CCL11 (eotaxin-1) and CCL26 (eotaxin-3), and the granulocytic markers Ly6G and CD11b (Figure 3). Interestingly, hepatic eosinophils presented lower levels of F4/80 and higher levels of Ly6G and CD11b than peritoneal ones (Figure 3A). They also expressed Sirpα (Figure 3A). Last, hepatic eosinophils expressed higher levels of CD162 and CD44 than eosinophils from the peritoneum (Figure 3A). On the contrary, eosinophils expressed very low levels of other molecules associated with myeloid antigen-presenting cells (macrophages or dendritic cells), such as Ly6C, MHCII, CD40, CD80, CD64, CD11c, CD4, or CD8 (Supplementary Figure 3). Of note, CCR2 was also expressed by peritoneal eosinophils (Figure 3B and Supplementary Figure 4).

### The Depletion of Eosinophils During Fasciolosis Leads to More Severe Clinical Signs and Liver Damage

**FIGURE 4:**
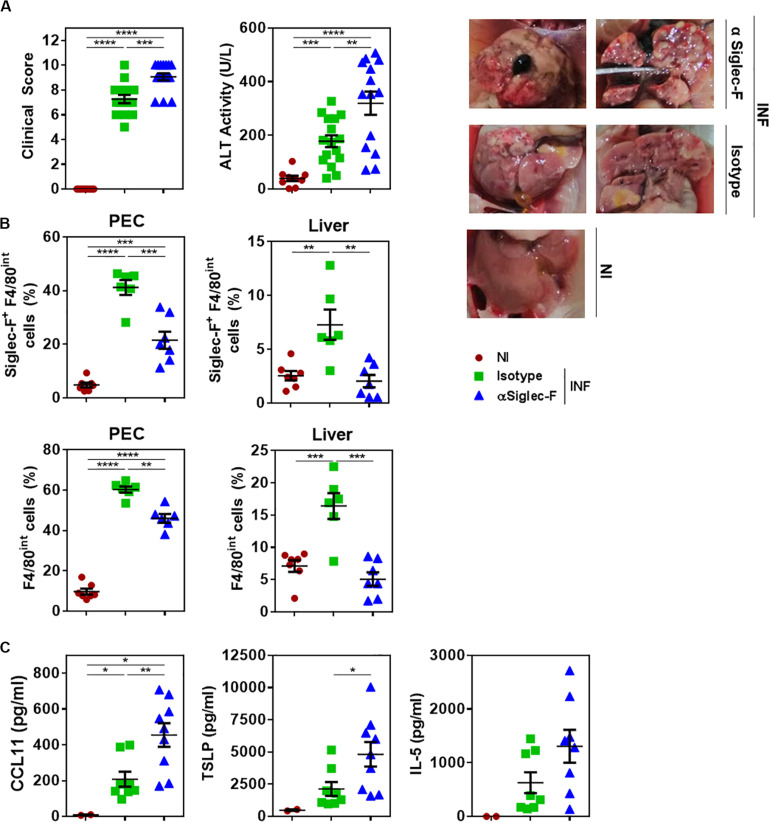
The depletion of eosinophils in infected animals leads to more severe clinical signs and increased liver damage. Mice received an intraperitoneal injection of 15 μg of monoclonal rat IgG_2*a*_ anti-Siglec-F antibody (clone E50-2440) the day before and after infection with *F. hepatica* and every 3 days until sacrifice. The control group consisted of mice injected intraperitoneally with isotype control antibody. Mice were sacrificed at day 20 post-infection and PECs and livers were removed. **(A)** Clinical score and ALT activity in sera in infected and non-infected mice. The results obtained for two independent experiments are shown (*n* = 15). On the right the livers obtained from the 3 groups are shown. **(B)** Frequency of Siglec-F^+^ F4/80^*int*^ (upper plots) or F4/80^*int*^ (lower plots) cells in the peritoneum of infected and animals by flow cytometry using anti-Siglec-F (clone S17007L) and -F4/80 (clone BM8) antibodies. Results obtained for one representative experiment out of two are shown (*n* = 7). **(C)** CCL11, TSLP and IL-5 levels in the peritoneal cavity of eosinophil-depleted and control infected mice determined by specific sandwich ELISA. Results obtained for one representative experiment out of two are shown (*n* = 9). Asterisks correspond to significant differences as follows: *p < 0.05, **p < 0,01, ***p < 0.001, ****p < 0.0001, performed by one way ANOVA followed by Tukey’s test.

We then analyzed the function of eosinophils during *F. hepatica* infection. To this end, infected mice were treated with a Siglec-F specific antibody during the infection to deplete eosinophils. As control, an IgG_2*a*_ isotype antibody was used. Interestingly, eosinophil *in vivo* depletion was associated with increased severity of clinical signs induced by *F. hepatica* infection and liver damage characterized by higher necrosis and fibrosis (Figure 4A). The depletion of eosinophils was confirmed by flow cytometry (Supplementary Figure 5A), both in PECs and livers of infected animals (Figure 4B and Supplementary Figure 5B). The decrease of eosinophil levels in PEC was associated with higher levels of CCL11, TSLP, and IL-5 in the peritoneum (Figure 4C), suggesting that infected animals increase the levels of these cytokines and chemokine to promote eosinophil differentiation during infection.

### Eosinophils Limit the Production of IL-10 by T Cells and Promote the Production of Th1 and Th2 Type Cytokines

**FIGURE 5:**
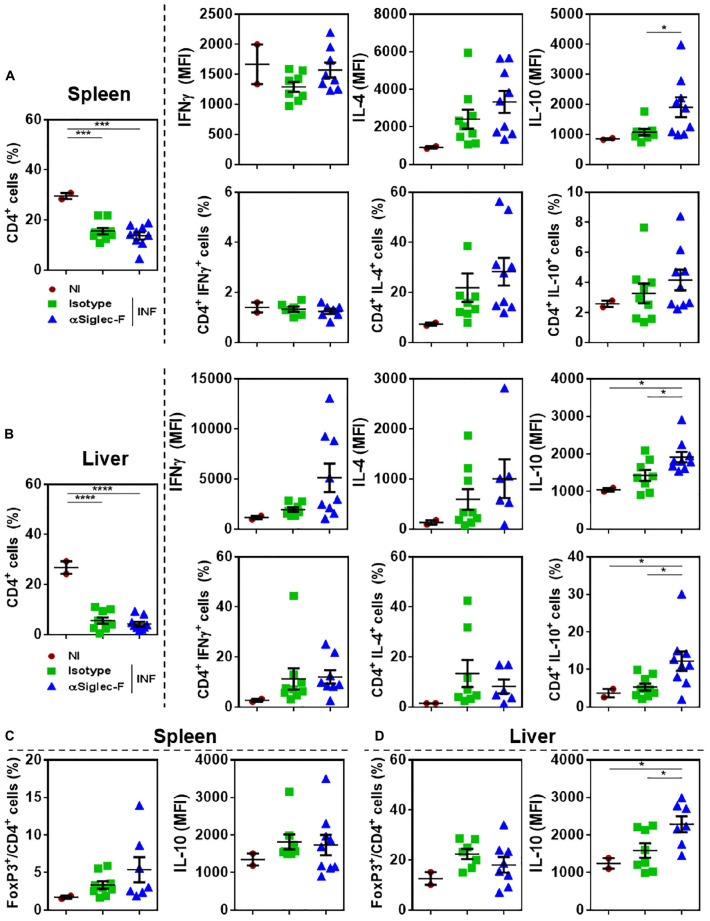
Eosinophil-depletion during fasciolosis leads to an increased production of IL-10 by hepatic and splenic CD4^+^ T cells. Mice (*n* = 7–8/group) received an intraperitoneal injection of 15 μg of monoclonal rat IgG_2*a*_ anti-Siglec-F antibody the day before and after infection with *F. hepatica* and every 3 days until sacrifice. The control group consisted of mice injected intraperitoneally with isotype control antibody. Mice were sacrificed at day 20 post-infection and spleens and livers were removed and analyzed by flow cytometry. **(A)** Frequency of CD4^+^ T cells in spleens from eosinophil-depleted and control infected mice and production of IFNγ, IL-4 and IL-10 by CD4^+^ T cells. **(B)** Frequency of CD4^+^ FoxP3^+^ T cells in spleens of eosinophil-depleted and control infected mice and production of IL-10 by CD4^+^ FoxP3^+^ T cells. **(C)** Frequency of CD4^+^ T cells in livers of eosinophil-depleted and control infected mice and production of IFNγ, IL-4 and IL-10 by CD4^+^ T cells. **(D)** Frequency of CD4^+^ FoxP3^+^ T cells in livers from eosinophil-depleted and control infected mice and production of IL-10 by CD4^+^ FoxP3^+^ T cells. Results obtained for one representative experiment out of two are shown (*n* = 9). Asterisks correspond to significant differences as follows: *p < 0.05, **p < 0.01, ***p < 0.001, ****p < 0,0001, performed by one way ANOVA followed by Tukey’s test.

To analyze the effect of eosinophil depletion during experimental fasciolosis, livers and spleens from infected mice treated with anti-Siglec-F or control antibody were removed and the T cell immune response was analyzed by flow cytometry (Supplementary Figure 6). Eosinophil depletion did not affect either the frequency or the cell number of splenic or hepatic CD4^+^ T cells of infected animals (Figures 5B,C and Supplementary Figures 6E,F). However, both spleen and hepatic CD4^+^ T cells from eosinophil-depleted infected animals produced higher levels of IL-10 than CD4^+^ T cells from control infected mice, while the production of IFNγ and IL-4 was not affected (Figures 5A,B). Splenic or hepatic regulatory CD4^+^ T cells, defined as FoxP3^+^ CD4^+^ T cells did not present any change either in frequency, cell number or IL-10 expression (Figure 5C and Supplementary Figures 6E,F).

**FIGURE 6:**
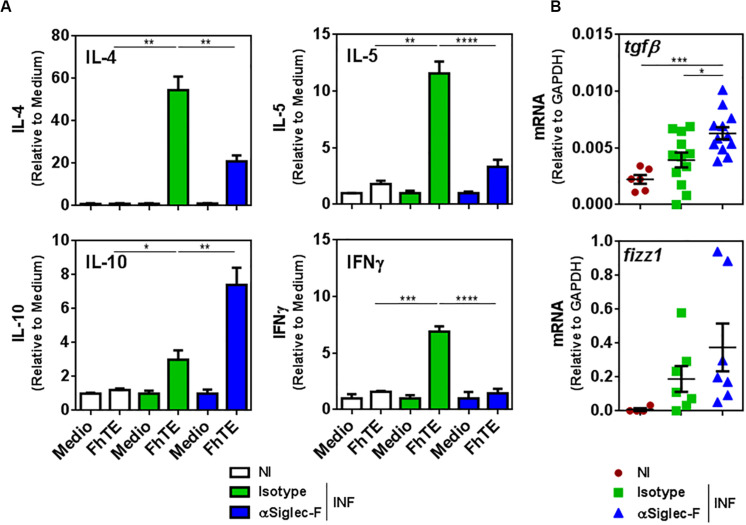
Eosinophils limit the production of IL-10 and TGFβ while promoting the production of IL-4, IL-5, and IFNγ. Mice (n = 7–8/group) received an intraperitoneal injection of 15 μg of monoclonal rat IgG_2*a*_ anti-Siglec-F antibody the day before and after infection with *F. hepatica* and every 3 days until sacrifice. The control group consisted of mice injected intraperitoneally with isotype control antibody. Mice were sacrificed at day 20 post-infection and spleens and livers were removed. **(A)** Splenocytes were cultured in the presence of FhTE (75 μg/mL) for 5 days at 37°C. Culture supernatants were collected and analyzed by ELISA for IL-4, IL-5, IL-10, or IFNγ. Results obtained for one representative experiment out of two are shown (*n* = 9). **(B)** *Tgf*β and *fizz1* gene expression was analyzed by qRT-PCR in livers from eosinophil-depleted and control infected mice. The results obtained for two independent experiments are shown (*n* = 15). Asterisks correspond to significant differences as follows: *p < 0.05, **p < 0.01, ***p < 0.001, ****p < 0.0001, performed by one way ANOVA followed by Tukey’s test.

In the same line, splenocytes from eosinophil-depleted animals stimulated with parasite-derived molecules (FhTE) produced higher levels of IL-10 with respect to splenocytes from control infected mice (Figure 6A). Moreover, the depletion of eosinophils during experimental fasciolosis was associated to a decreased production of IL-4, IL-5 and IFNγ (Figure 6A), suggesting that in the absence of eosinophils the Th2 or Th1 immune responses are impaired.

Last, the depletion of eosinophil induced higher gene expression of the immunoregulatory cytokine TGFβ and Fizz-1 in the livers of infected animals, although only the increase of TGFβ was significant (Figure 6B). Altogether, these results indicate that eosinophils limit the regulatory immune response and favor the Th2 or Th1 immune response, which is more effective to limit the damage induced by the parasite infection.

### Eosinophils Promote a Humoral Response That Is More Effective in Triggering Degranulation

**FIGURE 7:**
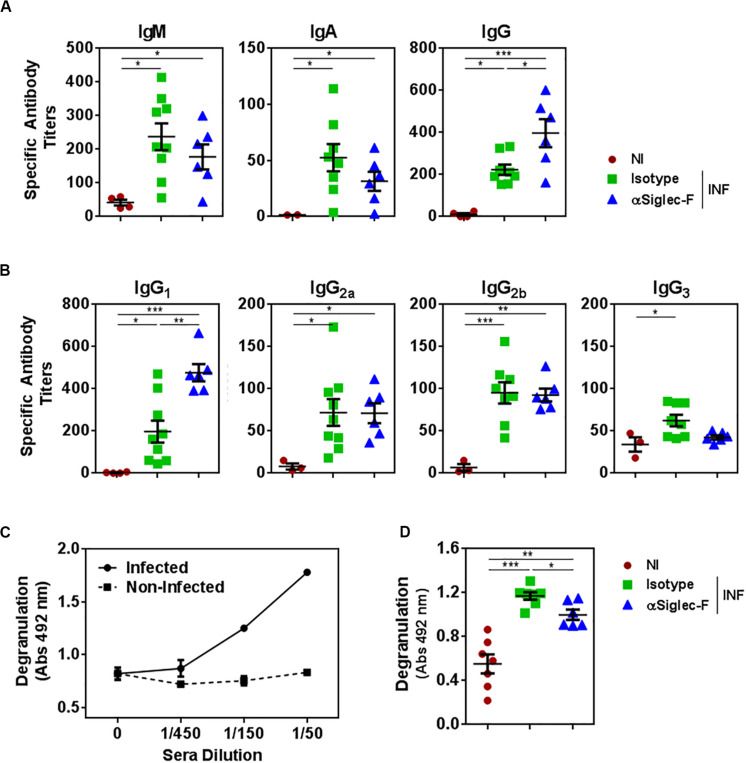
The presence of eosinophils promotes the production of antibodies with higher capacity to trigger degranulation. Mice (*n* = 7) received an intraperitoneal injection of 15 μg of monoclonal rat IgG_2*a*_ anti-Siglec-F antibody the day before and after infection with *F. hepatica* and every 3 days until sacrifice. The control group consisted of mice injected intraperitoneally with isotype control antibody. Mice were bled at 20 d.p.i. **(A)** Parasite-specific IgG, IgA, and IgG antibodies were detected by ELISA on FhTE coated plates. Results obtained for one representative experiment out of two are shown (*n* = 9). **(B)** Parasite-specific IgG_1_, IgG_2*a*_, IgG_2*b*_, and IgG_3_ antibodies were detected by ELISA on FhTE coated plates. **(C)** Degranulation of bone marrow derived-eosinophils (1 × 10^6^/well) from infected animals was evaluated by determining the eosinophil peroxidase enzyme liberation on eosinophils incubated in the presence of serially diluted sera from infected and non-infected mice with FhTE (100 μg/ml) in the presence of OPD and H_2_O_2_. **(D)** Sera (diluted 1/50) from eosinophil-depleted and control mice were incubated with bone marrow derived-eosinophils (1 × 10^6^/well) in the presence of FhTE (100 μg/ml), OPD and H_2_O_2_ for 3 h at 37°C. Degranulation was evaluated by determining the liberation of eosinophil peroxidase to the culture medium. Results obtained for one representative experiment out of two are shown (*n* = 9). Asterisks correspond to significant differences as follows: *p < 0.05, **p < 0.01, ***p < 0.001, ****p < 0.0001, performed by one way ANOVA followed by Tukey’s test.

In order to evaluate whether the depletion of eosinophil affected the humoral response induced during the infection with *F. hepatica*, we analyzed the antibody titers of parasite-specific antibodies. Eosinophil depletion did not alter the titers of parasite-specific IgM and IgA antibodies (Figure 7A). However, eosinophil-depletion induced a significant increase in the specific IgG antibody titers (Figure 7A), which was associated with an increase in IgG_1_ and a decrease of IgG_3_ antibodies with respect to the control infected group, while no changes were detected with IgG_2*a*_ and IgG_2*b*_ titers (Figure 7B).

Interestingly, the antibodies induced during the infection were capable of inducing degranulation of eosinophils, while antibodies from non-infected animals did not (Figure 7C). Furthermore, antibodies from control infected animals induced higher levels of degranulation than antibodies from eosinophil-depleted mice. Altogether, these results indicate that eosinophils modulate the effector mechanisms mediated by parasite-specific antibodies during fasciolosis.

## Discussion

In this study, we investigate the role of eosinophils during *F. hepatica* infection and show that they are beneficial to the host by promoting an effective immune response that partially controls liver damage. Accumulation of eosinophils is a well-documented feature of helminth infections since these cells can be vital to develop Th2 immune responses against parasitic helminths (22). However, their role during infection is rather controversial. Experimental infections either with the nematodes *Brugia malayi* (23), *Nippostrongylus brasiliensis* (24), *Angiostrongylus cantonensis* (25, 26), *Heligmosomoides polygyrus* (19), *Trichinella spiralis* (27), *Strongyloides ratti* (28), *Trichuris muris* (29), or the trematode *Schistosoma mansoni* (30) are characterized by strong eosinophilia. However, only in the case of *B. malayi* or *N. brasiliensis* infections, eosinophils seem to mediate unquestionable host protection by reducing damage caused by the parasite (17, 31–34). Indeed, different works on helminth infections have reported an irrelevant (19, 25, 29, 35–38) or even harmful (19, 39) role of eosinophils to the host.

First, we identified eosinophils during *F. hepatica* infection and showed that they augment with the time of infection, a feature that also characterizes infections by *B. malayi* (23), *N. brasiliensis* (24), *A. cantonensis* (25, 26). In line with our results, eosinophils constitute 40–60% of the cells in the peritoneal cavity of *B. malayi* or *S. mansoni*-infected mice (23, 30). The parasite itself could promote the accumulation of greater number of eosinophils in the peritoneal cavity after invading the intestinal wall that allows the juvenile flukes to reach the peritoneal cavity. However, this occurs in early stages of the infection (3). Moreover, eosinophils could accumulate in the peritoneum in response to the presence of immature parasites in the peritoneal cavity, even at 21 days post-infection (40, 41). However, we failed at detecting parasites in the peritoneum in our experimental infections in mice. Last, eosinophils could also derive from the blood due to portal hypertension produced by fibrosis in livers (42), since infected mice with severe clinical signs present hemorrhagic ascites which is a marker of advanced liver injury (43, 44). Thus, the eosinophils found in the peritoneal cavity could represent an attempt of the host to allow eosinophil recruitment to the liver to fight against the parasite.

Eosinophilia in the peritoneum was associated with high levels of essential factors for the development, recruitment, activation and maintenance of eosinophils, such as TSLP and IL-5 from the first week post-infection and CCL11 from the second week post-infection, reflecting a suitable milieu for eosinophil activation and differentiation (23). Indeed, the depletion or deficiency of IL-5 or CCL11 or blockade of IL-5R abrogates eosinophilia in several helminth infections (18, 23, 28, 38, 45–47). In addition, both hepatic and peritoneal eosinophils expressed CD44, CD162, and CD11b, molecules involved in cell adhesion during inflammatory conditions (48), indicating an activated state in these cells (49–51). Furthermore, according to the levels of these molecules, hepatic eosinophils seem to be more activated than peritoneal ones, a feature that could be associated with their effector mechanisms (52, 53). Also, the expression of high levels of Sirp-α by both hepatic and peritoneal eosinophils from *F. hepatica* infected mice might promote their survival, since it regulates their homeostasis, by regulating the degranulation process and inducing signals during inflammation (54). Last, the absence of MHC class II and co-stimulatory molecules rules out the possibility for eosinophils to act as non-professional antigen-presenting cells in the context of *F. hepatica* infection (55, 56).

The depletion of eosinophils during infection with *F. hepatica* partially protected mice from clinical signs and liver damage, indicating that they contribute to the defense mechanisms induced by the host. Indeed, our results strongly suggest that eosinophils would act by two complementary mechanisms: by limiting the production of IL-10 by CD4^+^ T cells and by promoting degranulation. Like other helminths, *F. hepatica* modulates the host immune response by inducing potent polarized Th2 and regulatory T cell immune responses, and by down-regulating the production of Th1 cytokines (5, 7, 57, 58). This immuneregulated environment favors the differentiation of regulatory T cells (7), a process that is mainly mediated by regulatory dendritic cells (4). Thus, a possibility is that eosinophils modulate dendritic cell function and limit the differentiation of Treg. Preliminary results from our laboratory suggest that eosinophils favor dendritic cell migration to the spleen, since a lower frequency of CD11c^+^ cells was detected in spleens from eosinophil-depleted mice (Supplementary Figure 7). Nevertheless, further data are necessary to determine whether dendritic cells from eosinophil-depleted mice have a higher capacity to differentiate Tregs. Furthermore, apart from limiting the production of IL-10 by T cells, eosinophils seem to favor the differentiation of Th2 type T cells, as judged by the strong production of IL-4 and IL-5 by stimulated splenocytes. The higher levels of IL-10 in eosinohil-depleted infected mice could be related to an increase of liver damage. In agreement with our results, EPO or major basic protein-deficient mice infected with *Litomosoides sigmodontis* present increased levels of IL-10 in the thoracic cavity, and a decrease in IL-5, which were accompanied by increased numbers of worms in infected mice (59, 60). Interestingly, IL-10 is associated with susceptibility of helminth infection (60) or with liver lesions caused by liver inflammation (61) or by helminth infection (62, 63).

On the other hand, eosinophils can directly injure or kill helminths by releasing a battery of toxic enzymes stored in their granules, although only *in vitro* evidences are available (64). In most cases, eosinophils require cooperation with antibodies or complement for killing capacity (65–67). Published results demonstrate that antibodies induced during *F. hepatica* infection can adhere to eosinophils and induce eosinophil degranulation in the presence of juvenile flukes (68, 69) by a mechanism where eosinophil proteins might be involved (70) or by eosinophil entrapment through extracellular traps (71). Our results highly suggest that eosinophils promote an antibody response that is more effective in mediating their degranulation. Mouse eosinophils are characterized by the expression of FcγRIII low affinity activating receptor that binds to IgG_2*a*_ and IgG_2*b*_ antibodies (72). The higher capacity of the sera from infected animals to induce degranulation was not associated with antibody titers since both groups had similar IgG_2*a*_ and IgG_2*b*_ titers. Thus, these antibodies might possess an advantage in terms of recognition of parasite antigens, although further experiments are required to confirm this hypothesis. Last, the higher IgG_1_ titers produced by eosinophil-depleted infected mice could be associated with the more severe liver damage that presented these animals, in an effort to contribute to parasite elimination, even though these antibodies do not seem to have a role in eosinophil degranulation. Of note, IgE would not play a role in eosinophil degranulation in *F. hepatica* infected-mice since the specific Fc receptors are not expressed on murine eosinophils (73).

In conclusion, our work demonstrates the beneficial immunomodulatory roles of eosinophils during *F. hepatica* infection by limiting IL-10 production and enhancing the capacity of specific antibodies to induce eosinophil degranulation, thus contributing to the understanding of eosinophil function during helminth infections.

## Data Availability Statement

All datasets presented in this study are included in the article/Supplementary Material.

## Ethics Statement

The animal study was reviewed and approved by Comisión Nacional de Experimentación Animal, CNEA, http://www.cnea.gub.uy/, National Law 18.611, Uruguay.

## Author Contributions

SF performed the experiments, analyzed the data, and drafted the manuscript. VC, MC, MF, ML, and SR-Z participated in the sample collection, cell culture, and flow cytometry analyses. SH and JT helped with the microscopy studies. TF conceived the whole work, designed the experiments, interpreted the data, and drafted the manuscript. All authors gave final approval of the version to be published.

## Conflict of Interest

The authors declare that the research was conducted in the absence of any commercial or financial relationships that could be construed as a potential conflict of interest.

## Abbreviations

ADCC: antibody-dependent cell cytotoxicity
Ig: immunoglobulin
IL: interleukin
MHC: major histocompatibility complex
TSLP: thymic stromal lymphopoietin.
