# Supplementary material for: Eosinophils Control Liver Damage by Modulating Immune Responses Against Fasciola hepatica
Source: Front Immunol. 2020 Sep 18;11:579801. doi: 10.3389/fimmu.2020.579801 (PMC7530260; doi:10.3389/fimmu.2020.579801)
Supplement: Supplementary file 1 [file Presentation_1.ppt]

## Slide 1
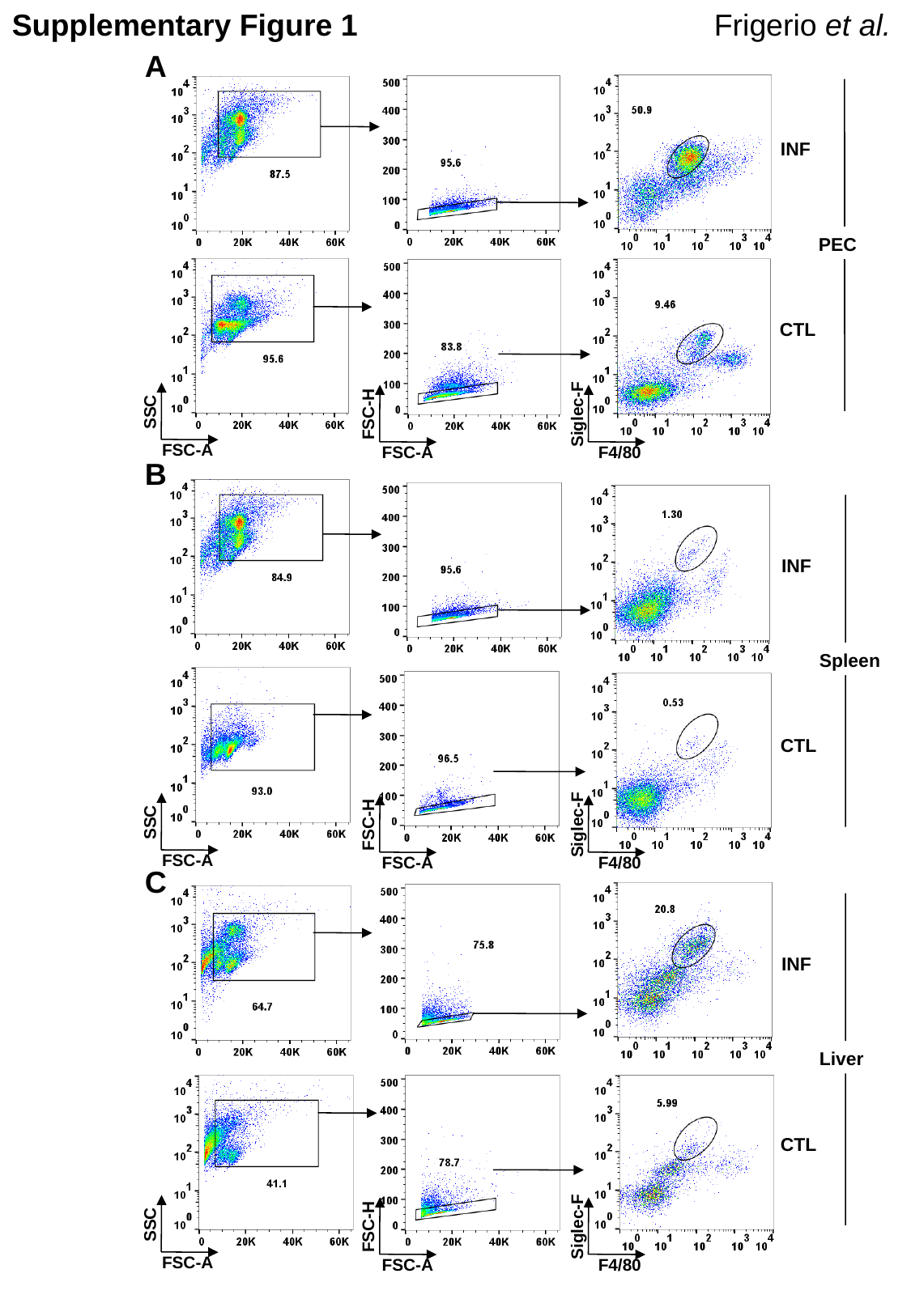

Supplementary Figure 1
Frigerio et al.
A
INF
PEC
CTL
SSC
FSC-H
Siglec-F
FSC-A
FSC-A
F4/80
B
INF
Spleen
CTL
SSC
FSC-H
Siglec-F
FSC-A
FSC-A
F4/80
C
INF
Liver
CTL
SSC
Siglec-F
FSC-H
FSC-A
FSC-A
F4/80

## Slide 2
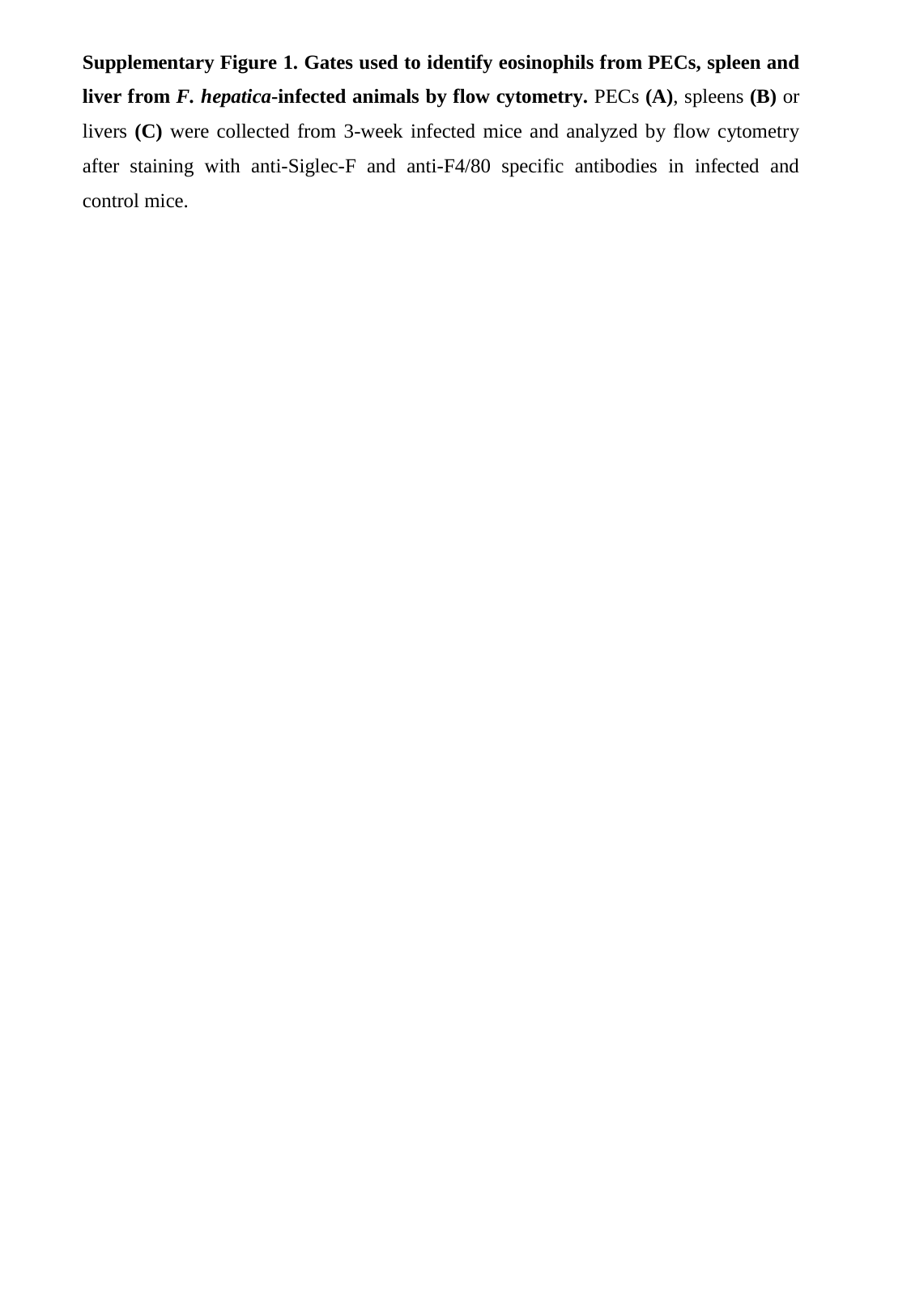

## Slide 3
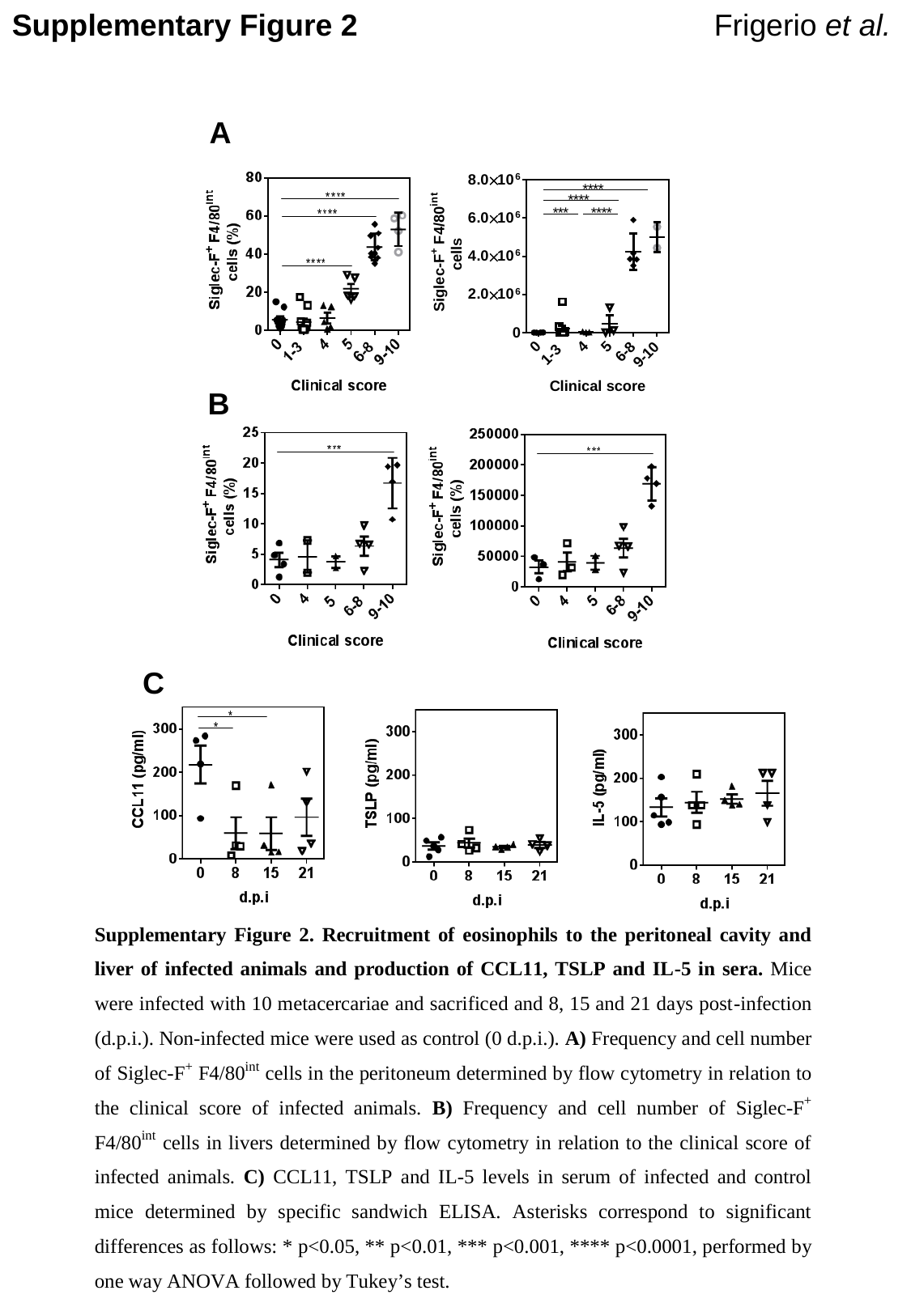

Supplementary Figure 2
Frigerio et al.
A
B
C

## Slide 4
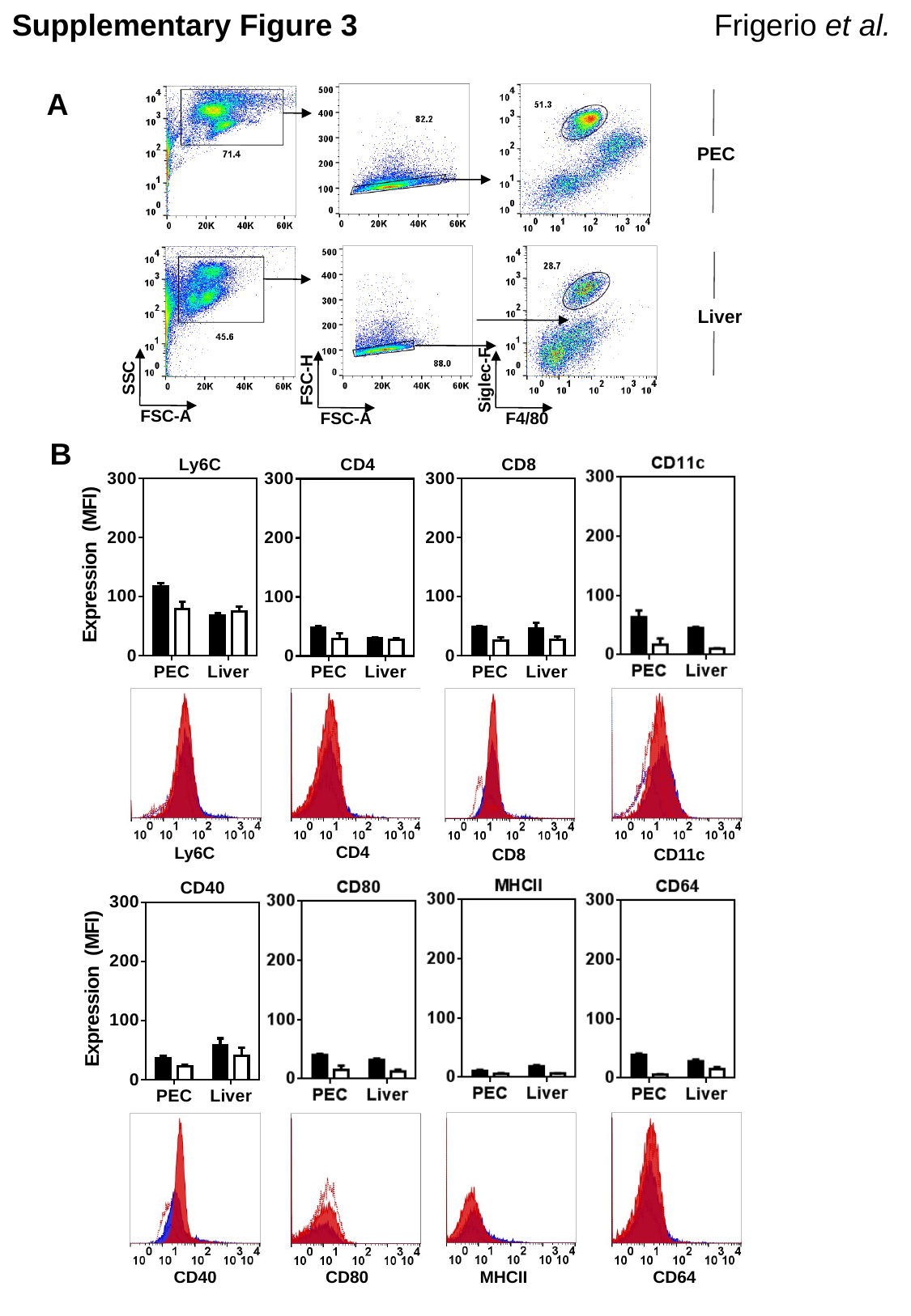

Supplementary Figure 3
Frigerio et al.
A
PEC
Liver
SSC
FSC-H
Siglec-F
FSC-A
FSC-A
F4/80
B
CD4
Ly6C
CD8
CD11c
CD80
CD40
MHCII
CD64

## Slide 5
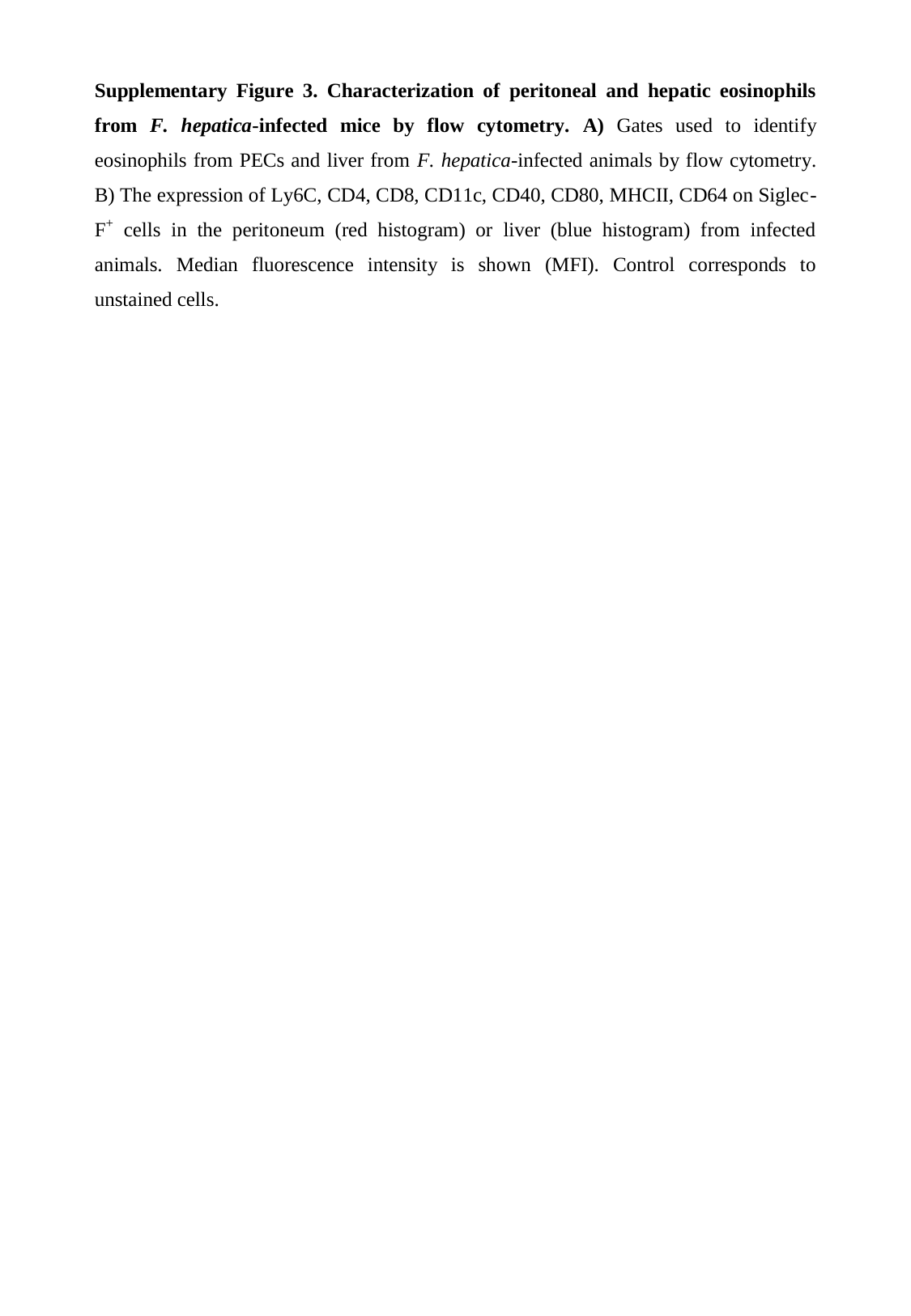

## Slide 6
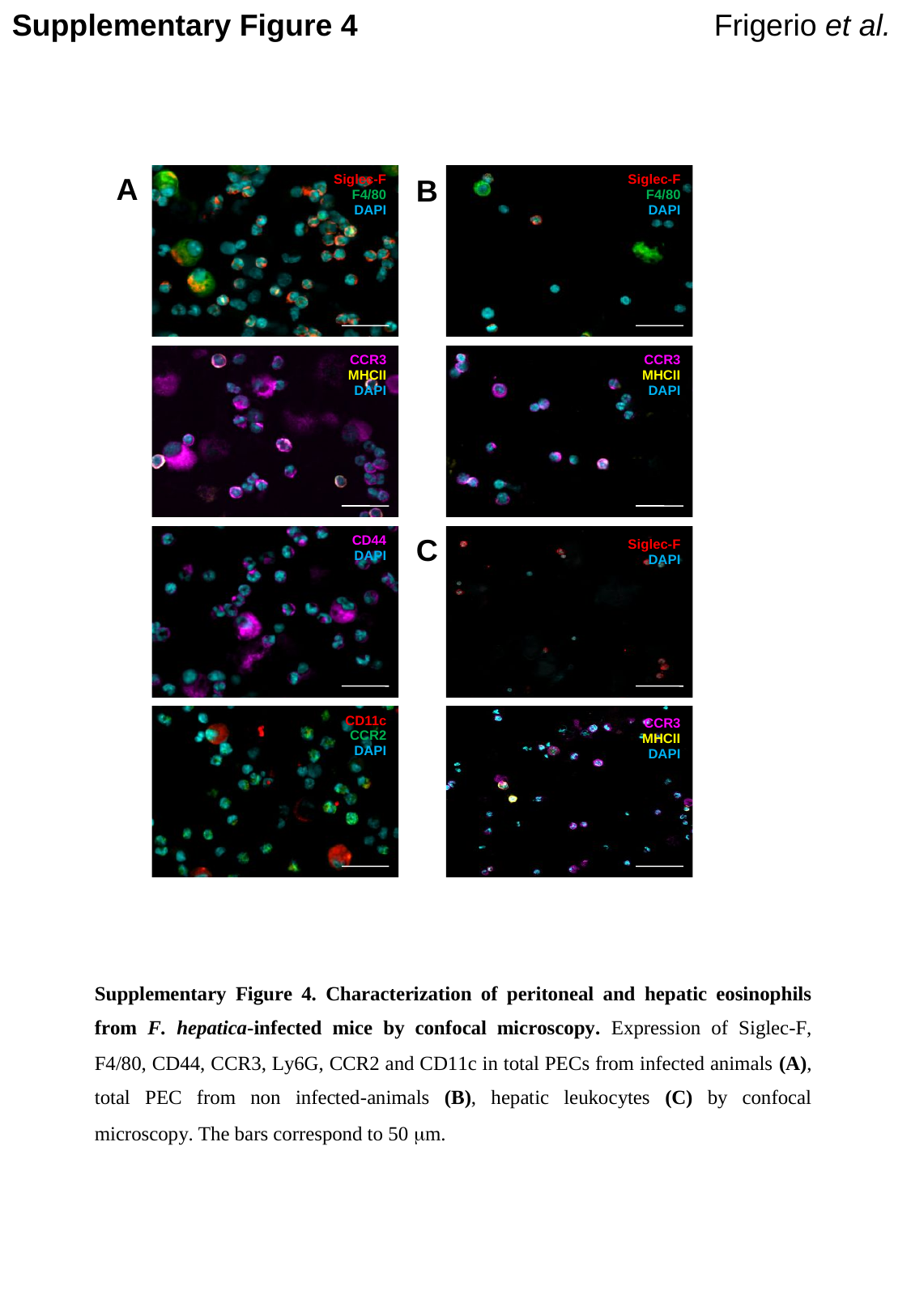

Supplementary Figure 4
Frigerio et al.
A
Siglec-F
F4/80
DAPI
B
Siglec-F
F4/80
DAPI
CCR3
MHCII
DAPI
CCR3
MHCII
DAPI
C
CD44
DAPI
Siglec-F
DAPI
CD11c
CCR2
DAPI
CCR3
MHCII
DAPI

## Slide 7
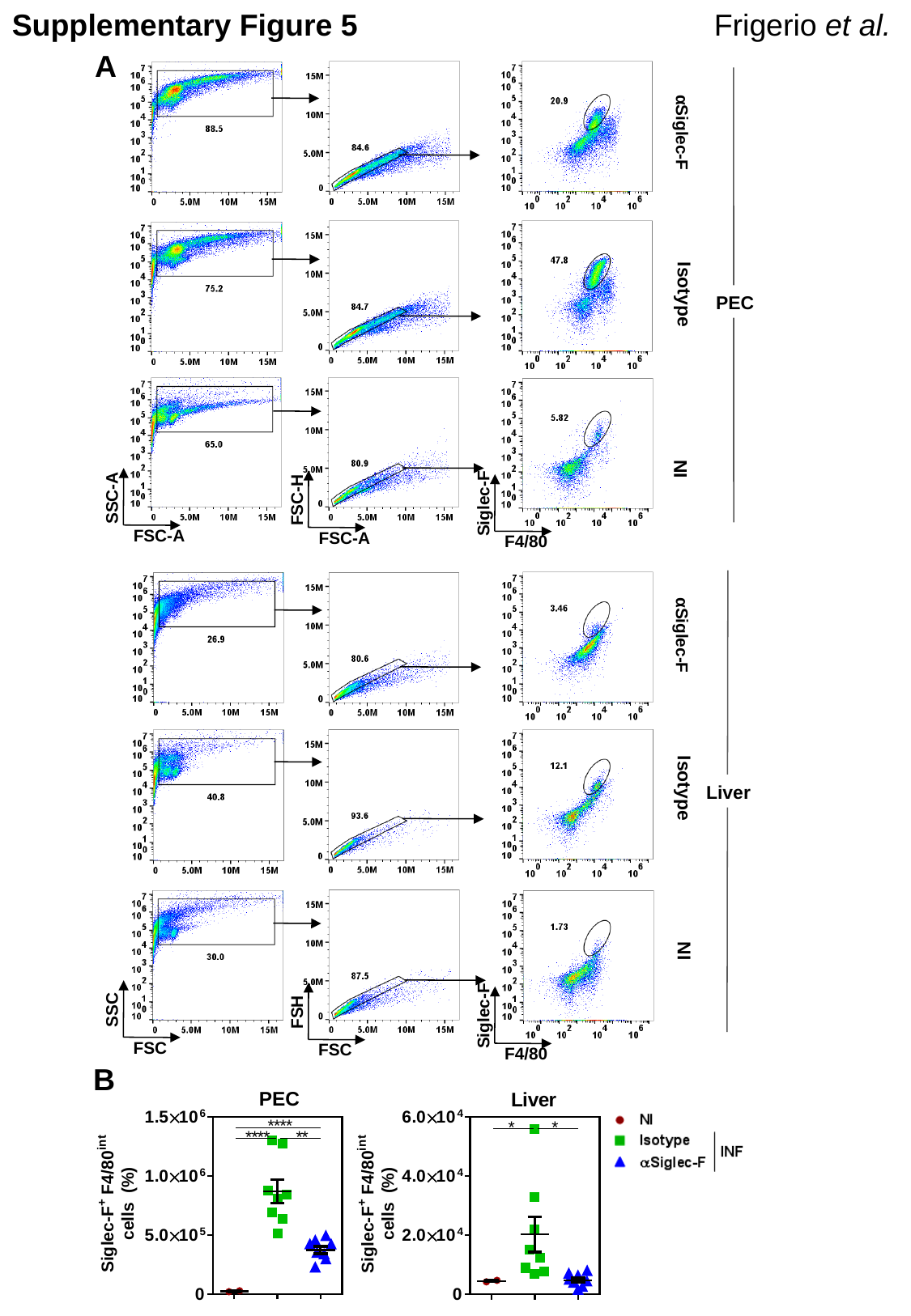

Supplementary Figure 5
Frigerio et al.
A
αSiglec-F
Isotype
PEC
NI
SSC-A
FSC-H
Siglec-F
FSC-A
FSC-A
F4/80
αSiglec-F
Liver
Isotype
NI
SSC
FSH
Siglec-F
FSC
FSC
F4/80
B

## Slide 8
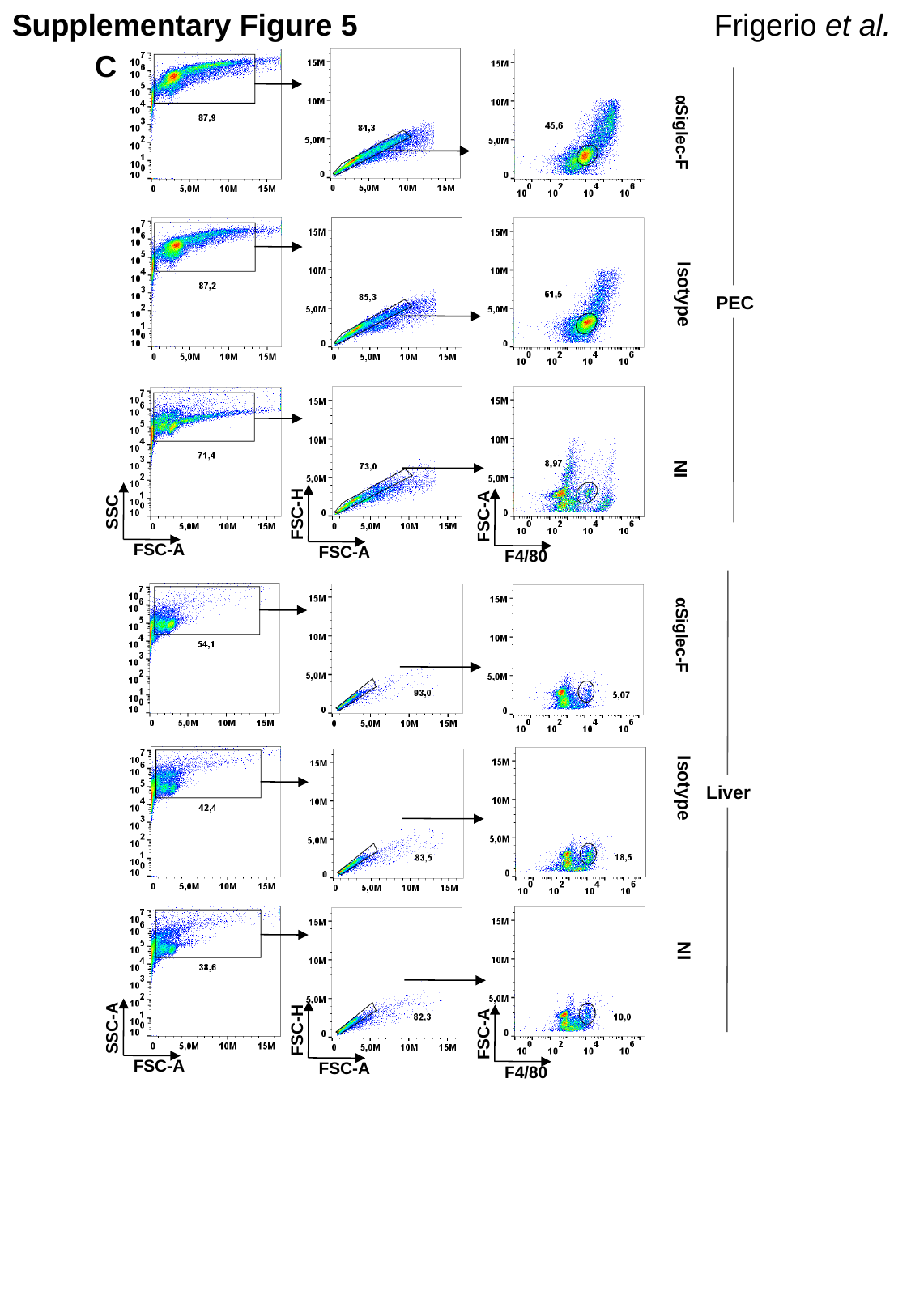

Supplementary Figure 5
Frigerio et al.
C
αSiglec-F
Isotype
PEC
NI
SSC
FSC-H
FSC-A
FSC-A
FSC-A
F4/80
αSiglec-F
Liver
Isotype
NI
SSC-A
FSC-H
FSC-A
FSC-A
FSC-A
F4/80

## Slide 9
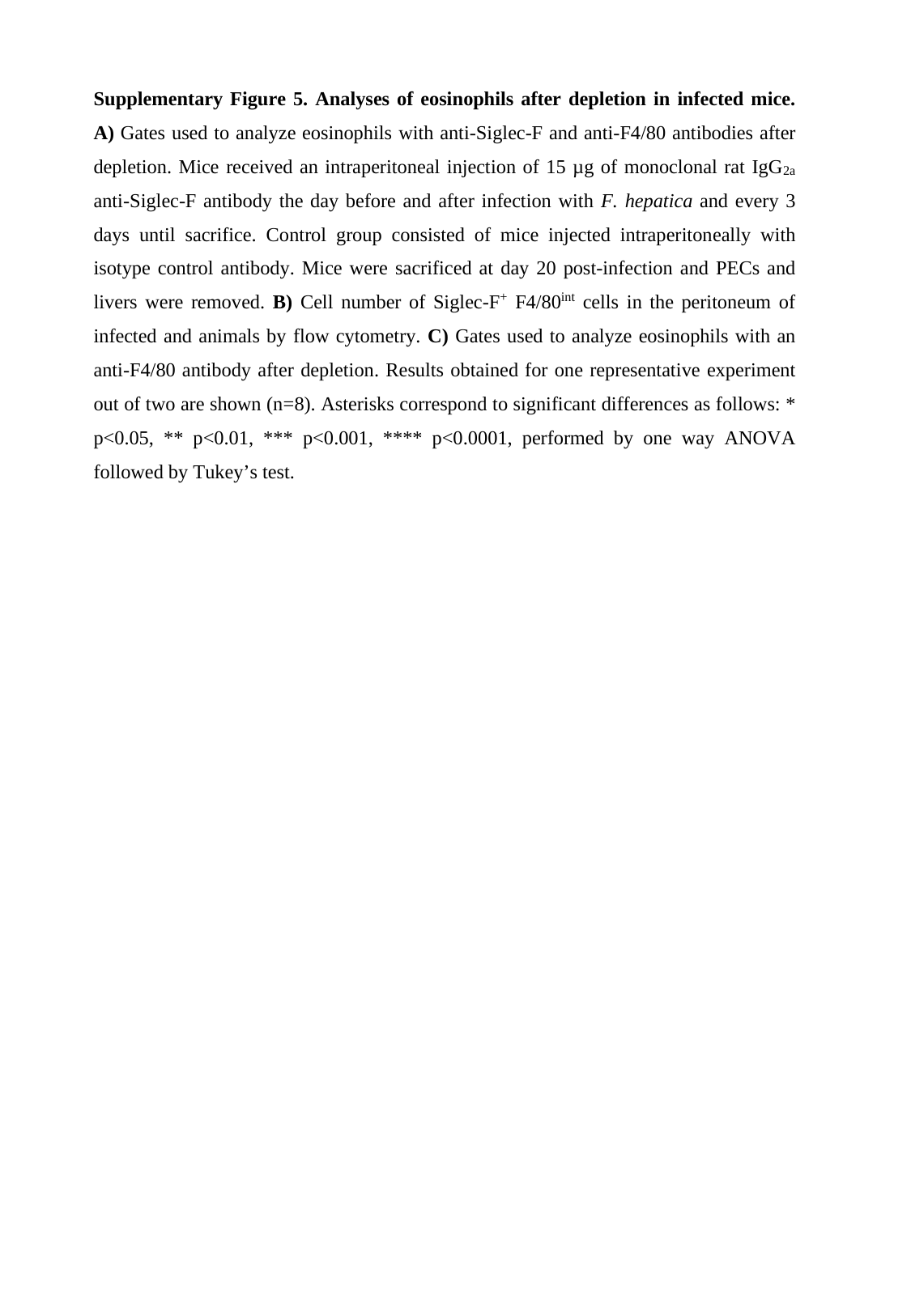

## Slide 10
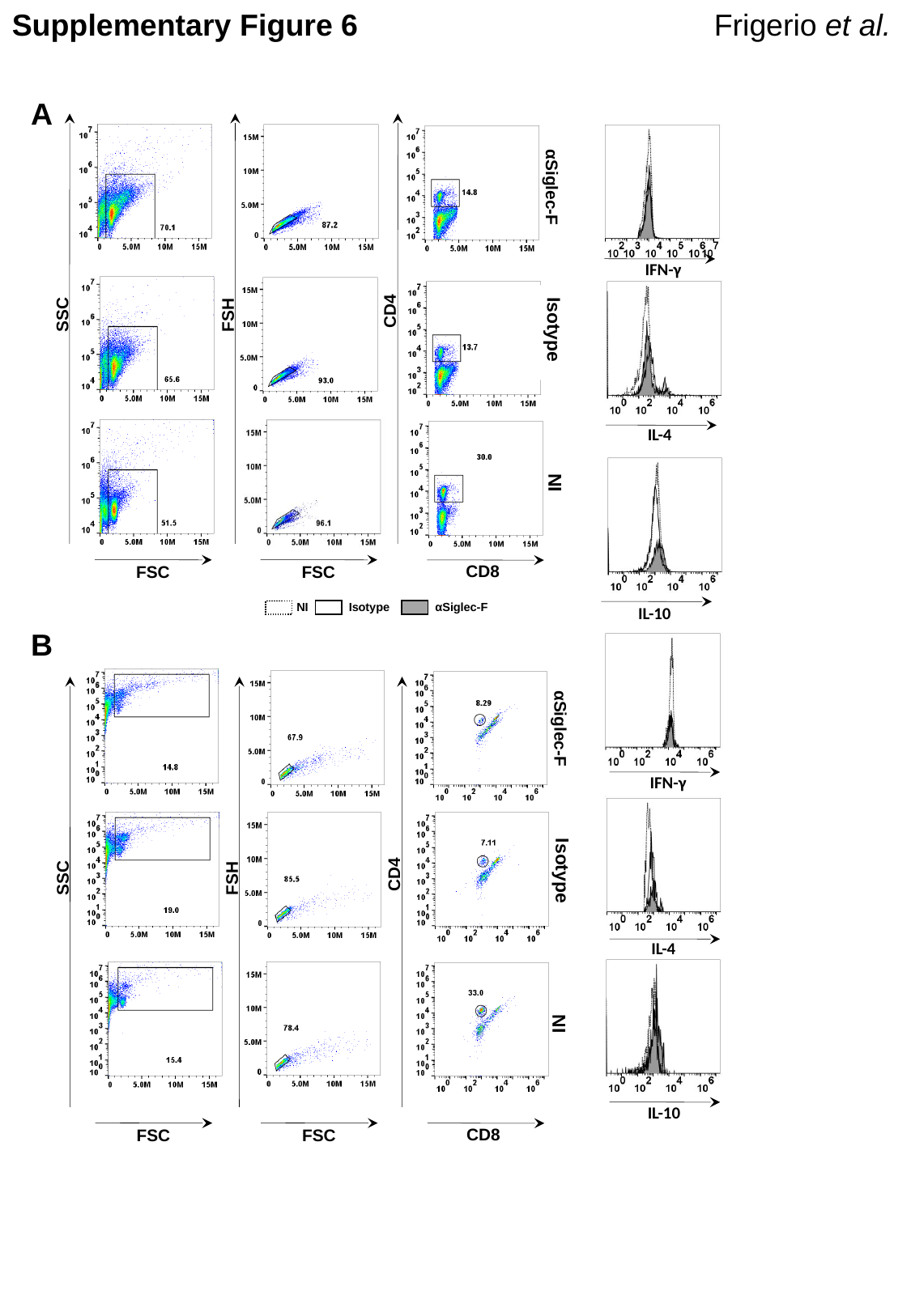

Supplementary Figure 6
Frigerio et al.
A
αSiglec-F
IFN-γ
CD4
SSC
FSH
Isotype
IL-4
NI
CD8
FSC
FSC
NI
 Isotype
 αSiglec-F
IL-10
B
αSiglec-F
IFN-γ
CD4
SSC
FSH
Isotype
IL-4
NI
IL-10
CD8
FSC
FSC

## Slide 11
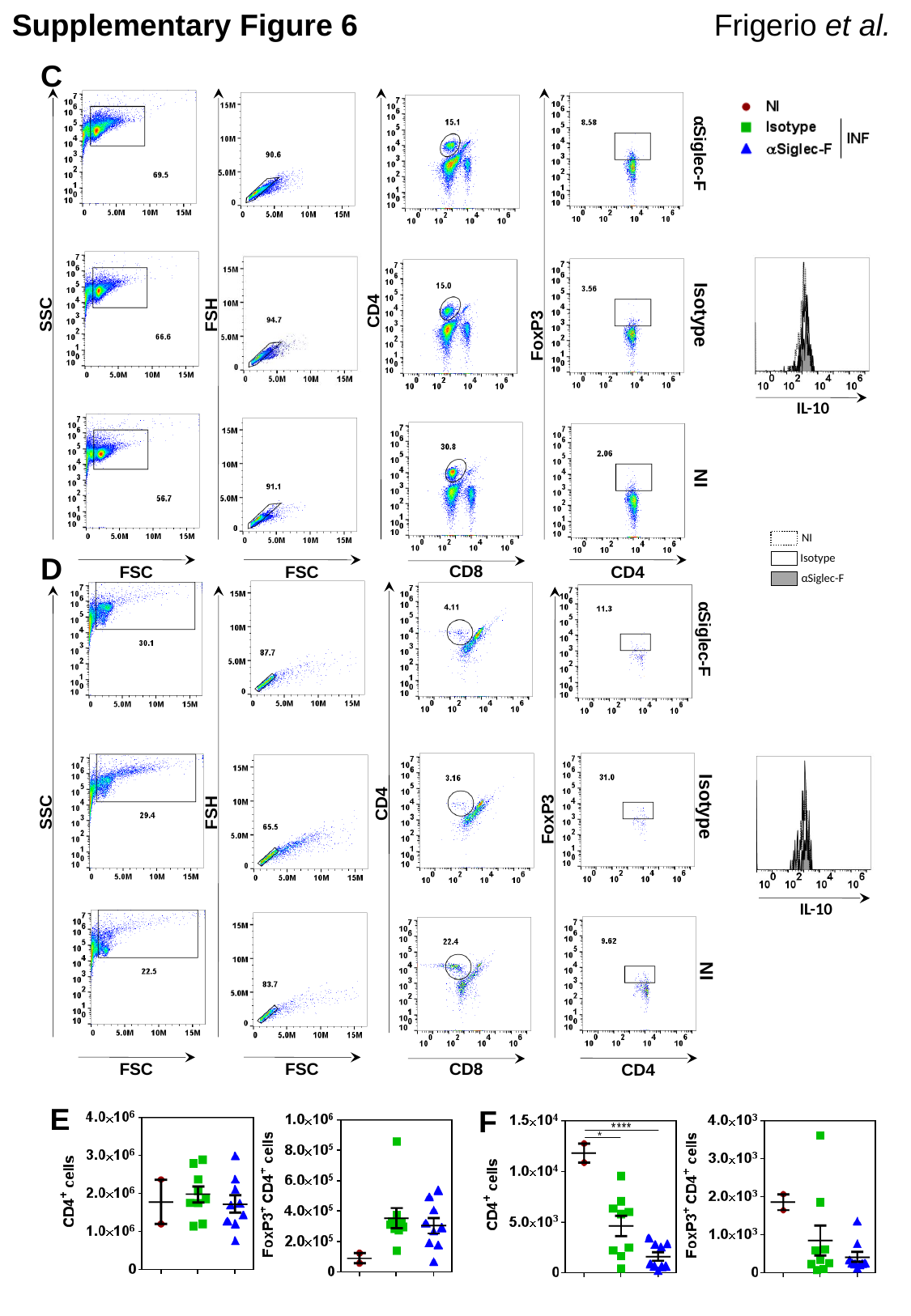

Supplementary Figure 6
Frigerio et al.
C
αSiglec-F
CD4
SSC
FSH
Isotype
FoxP3
IL-10
NI
NI
D
Isotype
CD8
FSC
FSC
CD4
αSiglec-F
αSiglec-F
CD4
SSC
FSH
Isotype
FoxP3
IL-10
NI
CD8
FSC
FSC
CD4
E
F

## Slide 12
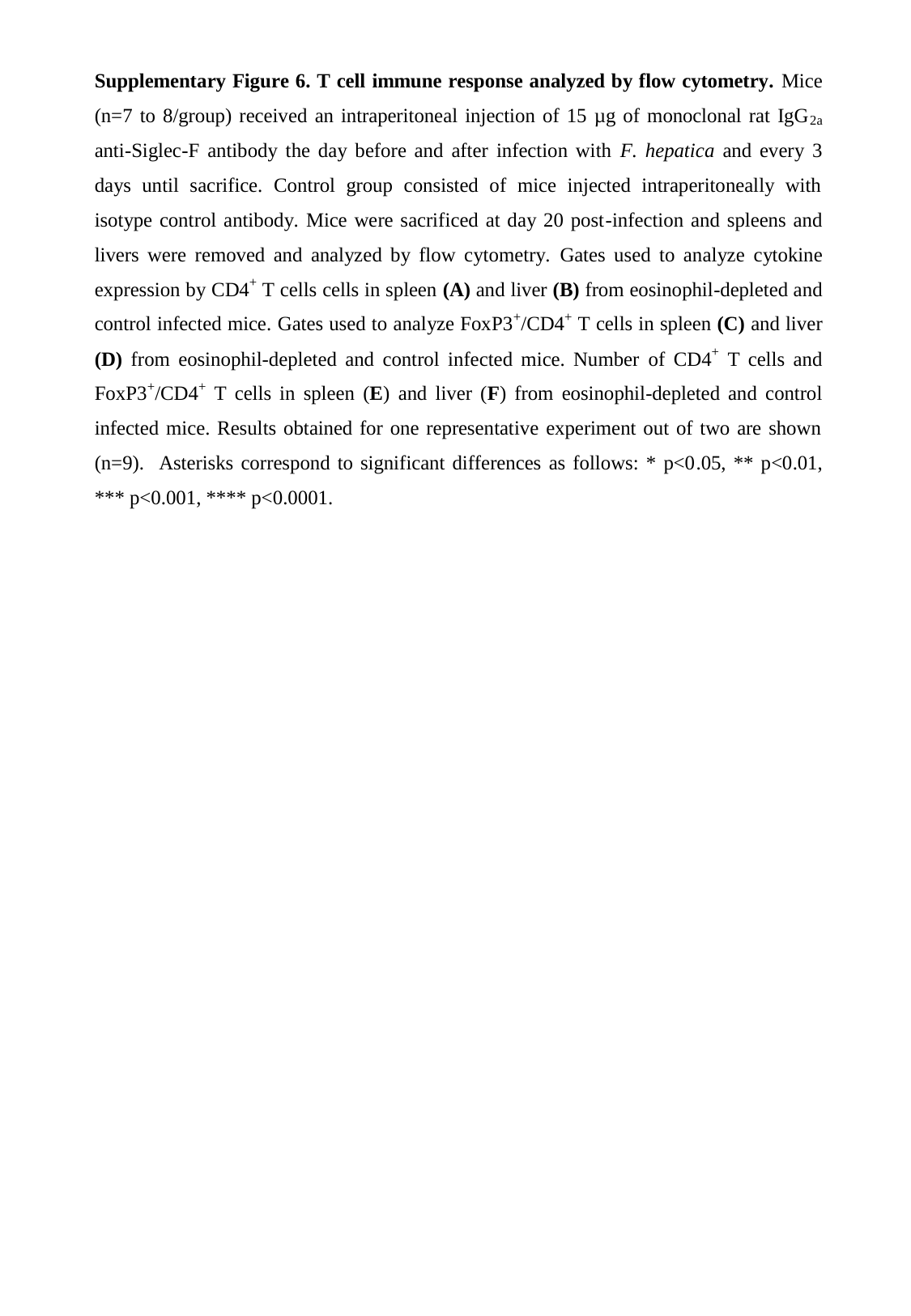

## Slide 13
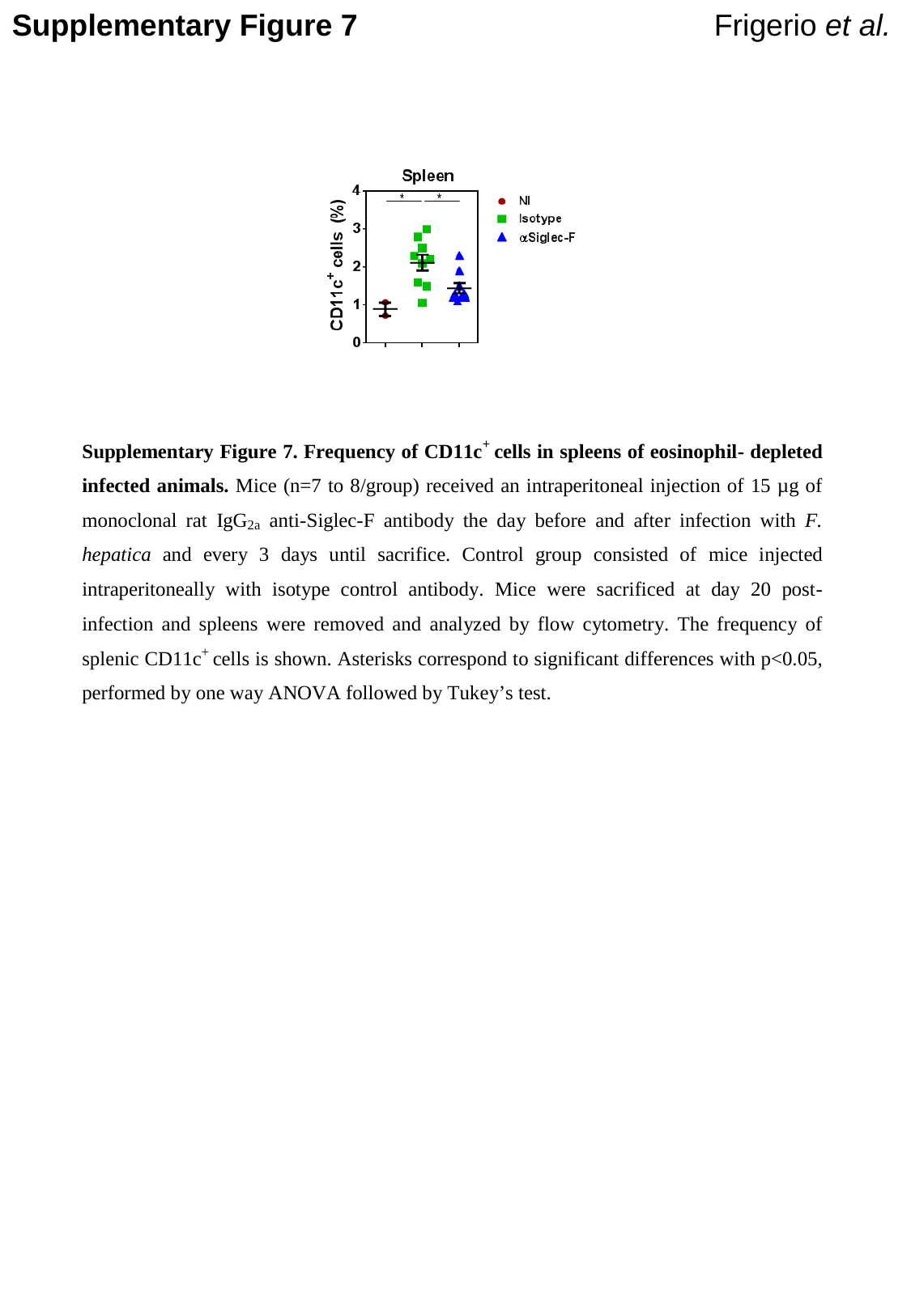

Supplementary Figure 7
Frigerio et al.
